# The mtDNA mutator mouse: Dissecting mitochondrial involvement in aging

**DOI:** 10.18632/aging.100109

**Published:** 2009-12-11

**Authors:** Daniel Edgar, Aleksandra Trifunovic

**Affiliations:** ^1^ Division of Metabolic Diseases, Department of Laboratory Medicine, Karolinska Institutet, S-14186 Stockholm, Sweden; ^2^ Cologne Excellence Cluster on Cellular Stress Responses in Aging-Associated Diseases (CECAD), University of Cologne, D-50674 Cologne, Germany

**Keywords:** mtDNA mutator mice, mtDNA point mutations, mtDNA deletions, oxidative deficiency, premature aging

## Abstract

The role of mtDNA mutations in aging has been intensely debated because of their low abundance and correlative connection with aging. The creation of mtDNA mutator mice provided the first evidence that somatic mtDNA mutations have the capacity to cause a variety of aging phenotypes in mammals, and they do so without inducing ROS production or increasing oxidative stress. We have recently provided evidence that the accumulation of point mutations in mtDNA leads to the synthesis of respiratory chain subunits with amino acid substitutions that impair complex stability in mtDNA mutator mice. Furthermore, we have demonstrated that the point mutations cause progressive respiratory chain deficiency, which, we propose, leads to premature aging. These results have been challenged by another group working on a similar model, who argues that the point mutations in mtDNA we found at very high levels in mtDNA mutator mice do not cause the phenotype. Instead, they argue that circular mtDNA molecules with large deletions, are the culprit. This intense debate about molecular mechanism of mitochondrial dysfunction that is causing progeroid phenotypes in the mtDNA mutator mice is the main topic of this research perspective.

## Introduction

Evidence from many paths of gerontological research suggests that mitochondria are one of the key players in aging. Not only do the number of mitochondria decrease in postmitotic tissues like heart, skeletal muscle and brain during aging [1,2], but a number of age-associated structural changes of mitochondria has been reported as well [1,3]. The most common aging-associated structural changes of mitochondria are alterations of cristae structure, but also matrix vacuolization or densification and a general enlargement of mitochondria have been found to increase with age [4-6]. Mitochondrial DNA (mtDNA) point mutations and deletions have been found in aging humans, and in many cases, have been correlated to mitochondrial dysfunction. Higher levels of mtDNA deletions were detected in postmitotic tissues e.g., skeletal muscle, heart and brain in contrast to kidney, spleen, skin and liver that are composed of dividing cells, indicating a tissue-specific "pattern" of deletion accumulation [7]. However, their possible causative effects have been intensely debated because of their low abundance (under 1% of deleted mtDNA molecules in whole tissue homogenate) and purely correlative connection with aging [8]. A real breakthrough occurred when it was discovered that mitochondrial dysfunction is unevenly distributed in tissues and that mtDNA point mutations and deletions accumulate in some single cells. For instance, the analysis of five *tRNA* genes showed high levels of clonally expanded mtDNA point mutations (49%-94%) in COX-deficient skeletal muscle fibers isolated from aged individuals [9]. In addition, point mutations associated with the major regulatory region for replication of mtDNA were found in a high proportion (up to 50%) in skin fibroblasts or skeletal muscles of individuals above 65 years of age [10,11].

To test the causative role of mtDNA mutations in aging we have developed the mtDNA mutator mouse that accumulates high levels of point mutations due to a proofreading deficiency of the mitochondrial DNA polymerase (POLG) [12]. Subsequently, a very similar model was developed by another group [13]. The two mouse models show basically the same phenotypes, differing only in the time of onset of phenotypes. In our hands, mtDNA mutator mice are born in Mendelian proportions, without any visible defects, but after 6-7 months they start to display a range of premature aging phenotypes, such as a weight loss, reduced subcutane-ous fat, alopecia, kyphosis, osteoporosis, anaemia, reduced fertility, heart disease, sarcopenia, progressive hearing loss and decreased spontaneous activity [12]. Their lifespan is also greatly reduced compared with wild-type littermate controls and they die at around 46 weeks of age [12].

In addition to the standard circular chromosome, mtDNA mutator mice harbour large linear mtDNA molecules caused by replication stalling [14]. These molecules are around 11-12 kb in length, and encompass the region between the origins of replication for the heavy (O_H_) and the light strands (O_L_). Around 25-30% of mtDNA consists of these linear molecules with deletion, and this ratio does not change as the animal age [12]. As these molecules exist at quite high levels, it has been proposed that this may also contribute to the progeroid phenotype of the mtDNA mutator mouse [12,14]. The POLG exonuclease activity may be involved in the resolution of replication intermediates at O_L_, which can explain why exonuclease deficiency will lead to replication stalling, thus leaving the mtDNA molecule susceptible to breakage at the stall site [14]. It has also been suggested that the time needed for the replication of mtDNA could be prolonged in the mtDNA mutator mouse, as evidenced by a high amount of replication intermediates [14]. This prolonged replication is proposed to exhaust resources in the mitochondria. Although plausible, this hypothesis still requires direct experimental verification [14].

The main consequence of the mitochondrial proof-reading deficiency in mtDNA mutator mice is the progressive, random accumulation of mtDNA mutations during the course of mitochondrial biogenesis.

**Table 1.** The number of mtDNA point mutations/10000 base pairs detected by different groups using the cloning and sequencing (CS) and the random mutation capture approach (RMC).

Genotype	Brain ~8 weeks	Heart ~25 weeks
WT	0,33(CS)[12] 0,0066 (RMC)[15]	4,58 (CS)[12] 2,11 (CS)[13]
HZ	1,33 (CS) [12] 3,3 (RMC) [15]	- -
MUT	9 (CS)[12] 14 (RMC)[15]	14 (CS)[12] 10 (CS)[13]

Different approaches have been used to measure levels of somatic mtDNA mutations and the results often vary considerably depending on the method used, e.g. cloning-sequencing (CS) or random mutation capture (RMC) (Table 1.). Both of these methods seem to detect similar differences in levels of mutations in mice heterozygous (Polg^+/mut^) or homozygous (Polg^mut/mut^) for the mtDNA mutator allele. However, the RMC method detects a 500-fold less mtDNA mutation load in control mice, i.e. wild type animals obtained after intercrossing mice het erozygous for the mtDNA mutator allele (Table 1.). The CS method is the most commonly used procedure to estimate the level of mtDNA mutations and involves PCR amplification of mtDNA, followed by cloning and sequencing of the obtained fragment. A problem with this method is that PCR might introduce mutations in the initial step, which are then carried through to the sequencing reaction. The RMC was developed by Vermulst et al. [15] and is based on the fact that mtDNA mutations will alter a given restriction enzyme recognition site. After restriction enzyme digestion only mutated molecules should be amplified by PCR and those are subsequently quantified and sequenced. However, the target sequence analyzed by this method is only 4 bases long (*Taq I* recognition site) and may therefore not represent the rate of mutations over the entire molecule [15]. The RMC assay appears to work very well on large sample sizes when the mutations are evenly distributed throughout the genome [16]. However, when existing mutations are few but have been amplified, they might be missed. MtDNA mutations can clonally expand in certain tissues, i.e. when a stem cell gives rise to many cells. This can also happen randomly when mtDNA molecules are segregate-ed or replicated unevenly.  In human colonic mucosa (a tissue known to clonally expand mtDNA  mutations) the RMC routinely detects between 10-1000 times less mutations than the CS approach and around 5 times less than with single-molecule-PCR based method [16].

The analysis of mtDNA mutation load in wild-type animals will be complicated when the animals are derived from a mother heterozygous for the mtDNA mutator allele. The reason is that the primordial germ cells of a heterozygous mother contain one copy of the mutant POLG allele and this could cause accumulation of a small amount of mtDNA mutations during their proliferation and maturation into oocytes. A genetic bottleneck occurs after the oocyte is fertilized whereby a small subset of all mtDNA molecules are partitioned into the primordial germ cells of the next generation [17]. Subsequent rounds of replication in the wt embryo are then carried out by only wt POLG, whereas in the mtDNA mutator embryo proofreading-deficient POLG is used. As a result, the embryo with the mutator allele will continue to introduce new mutations, whereas the wt embryo will amplify only the pre-existing mutations with high fidelity. This may explain why wt littermates of mtDNA mutator mice may have a moderately increased mutation load in comparison with other wild-type mice whose mitochondria have never come in contact with the mtDNA mutator allele. Heterozygous animals from the same cross might continue to accumulate random mtDNA mutations, but to a much lesser extent than mtDNA mutator mice. The pedigree of the analyzed wild-type mice is therefore an important factor to consider when determining mtDNA mutation loads.

In order to resolve this issue, we believe that a direct cloning and sequencing approach should be used. This would involve the cloning of large fragments or an entire mtDNA molecule into a vector, without the use of an intermediate PCR step [18]. In this way PCR-induced errors will be avoided and a more complete picture of the distribution of mutations on the population of mtDNA molecules will be achieved. Direct cloning and sequencing has 3 major drawbacks. First, it is labour intensive. Second, it would require a large sequencing effort to sample the same magnitude of bases as a RMC assay, making sequencing costs prohibitive. Finally, cloning of small amounts of sample DNA is often inefficient and studying single cells is therefore not possible at the moment. Alternatively, the cloning step could be circumvented by usage of newly developed deep sequencing techniques that could be quantitative simply because the same region of the mtDNA would be sequenced over and over again. However, many deep sequencing methods have inherent error rates, which may obscure somatic mtDNA mutation loads.

In a recently published paper, Loeb and co-workers  suggest that a third type of mtDNA mutation, besides the very abundant point mutations and linear deletions we have reported [12] are the main driving force behind the shortened lifespan in mtDNA mutator mice [19]. This third type of mutation is circular mtDNA molecule with large deletions of several thousand base pairs. The main argument for this conclusion is that the RMC method detects a 500-fold increase of mtDNA point mutations in heterozygous, Polg^+/mut ^in comparison with wild-type mice, despite the fact the heterozygous mice have a normal lifespan [15]. The authors also reported that circular mtDNA molecules with deletions are highly increased in frequency throughout the lifetime of the mtDNA mutator mouse. Similar molecules are also found to accumulate in the wt and Polg^+/mut^ mice , but to a much lesser extent (10- 100 times lower). In light of this evidence they conclude that the circular mtDNA molecules with deletions correlate much better with the phenotype and therefore must be the driving force behind the premature aging of mtDNA mutator mice [19]. However, their study is purely correlative and it does not take into account threshold levels that are the imperative in mitochondrial genetics. In their study, they have estimated only the relative amount of circular mtDNA molecules with deletions between genotypes. To estimate the importance of these circular mtDNA molecules with deletions it is necessary to know what fraction of the total mtDNA they represent.

We recently attempted to detect these deletions using long extension PCR technique.  As controls, we used two strains of mice with known amounts of deletions, the mito-mouse and the deletor mouse [20,21]. These mouse strains carry circular mtDNA molecules with single (mito-mice) or multiple (deletor-mice) deletions in their mitochondria. Although we have robustly detected deleted mtDNA molecules in serial dilutions of these control samples we were not able to detect any deletions in mtDNA mutator heart or liver samples. Recently, Kraytsberg et. al used single molecule long extension PCR to detect deletions in mtDNA mutator samples provided by Loeb and co-workers [22]. They did not detect deleted mtDNA among more than 320 amplified molecules from duodenum and 144 molecules from heart of mtDNA mutator mice [22]. This completely agrees with our data obtained from heart and liver of mtDNA mutator mice. Kraytsberg et. al detected some mtDNA deletions in brain tissue, but estimate the relative levels to be around 1% and they argue that this could be an overestimation [22].

It is unclear how these rare circular molecules with deletions could be the major driving force behind the shortened lifespan in the mutator mice. The mitomice with deletions in up to 30% of all mtDNA molecules do not die early, whereas those with deletions in 70% suffer from renal failure [23]. The deletor mouse, although generating multiple deletions of the same range as proposed for mtDNA mutator mice, show no premature aging phenotype.  The deletor mice have normal lifespan but develop mitochondrial myopathy late in life [21]. Furthermore, deletions are not related to Parkinson's disease until they reach a threshold of around 52% in dopaminergic neurons of humans [24]. The same is true for most diseases caused by mutations in mtDNA. There is always a threshold level of mutation that has to be reached before the mutation causes respiratory chain dysfunction. This threshold level will vary depending on mutation, but is generally between 60% (for deletions) - 90% (point mutations) [25]. In the mtDNA mutator mouse the mutations are spread out over the genome and a very high threshold level of a particular mutation might not be reached. However, if every molecule accumulates approximately 20 mutations, many of these will be in protein coding genes. For COXI for example there is an 88% chance that the gene contains at least 1 mutation in a specific mtDNA molecule and a 61% chance that it contains at least 2 mutations of which 40% will cause amino acid change. Then there are another 12 genes for which numbers are slightly different depending on the size of the genes. Assuming random distribution of the mutations, a threshold level will be reached easily, not by one specific mutation, but by the sum of all different mutations. Deletions, though affecting many base pairs, still need to be at significant levels to cause mitochondrial dysfunction. It has been estimated that there is about 1000 point mutations for every single deleted mtDNA molecule in mtDNA mutator mice (if there is >0.02 deletion per mtDNA molecule) [22]. This also means that mtDNA mutator mice have about 800 additional point mutations for every additional deletion when compared to Polga^+/mut^ animals [22].

Loeb and co-workers do not report increased amounts of deletions in COX-negative cells (cytochrome c oxidase deficient cells - indicating mitochondrial dysfunction), only an increase in point mutations [19]. Large circular mtDNA deletions typically span over several protein coding genes and include a number of *tRNA* genes. Therefore, consequences of the large deletions could be detected as a reduction in mitochondrial transcript levels or as a decrease in translation of mitochondrially-encoded proteins. This, however, does not seem to be the case. We found that the pattern of transcriptional change was more consistent with increased activity from the heavy strand promoter, rather than a loss of components via deletions. Translation was also normal, but we did observe increased protein turnover [26]. So the mechanism by which these rare, circular mtDNA molecules with deletions would be causing the premature aging phenotype remains unclear.

So what have we learned from the mtDNA mutator mouse? Are mtDNA mutations relevant in normal aging? Clearly wt mice never accumulate the same level of point mutations as mtDNA mutator mouse. They do, however, acquire mitochondrial dysfunction with old age, as do the mtDNA mutator mice. This indicates mitochondrial dysfunction as not only being correlated to aging, but also causative. Results from mtDNA mutator mice clearly show that mutations in mtDNA *can* cause problems that resemble premature aging. Of these mutations, the amino acid changing mutations in protein coding genes seem to be the most deleterious [26]. This is in agreement with a recent finding than non-synonymous mutations that change amino acids in respiratory chain subunits are strongly selected against and very rarely emerge in future generations, whereas tRNA and rRNA mutations seemed to have less severe functional consequences and could easily be inherited [27]. In addition to loss of function mutations there could be dominant deleterious mutations, like the C>T mutation at nucleotide 5545 of mtDNA that cause pathology at very low levels [28]. One attractive candidate for the failure of the organism to thrive comes from the finding that stem cell function in the mtDNA mutator mice seems to be altered (Ahlquist et al. unpublished results). On the organismal level, it remains to be determined what type of changes and in which cells have the most important effects. Additional studies on mtDNA mutator mice in conjunction with old control mice might be able to dissect out which changes are important and which are just correlative in the way mitochondria affect the aging process.

### ACKNOWLEDGMENTS

A.T. is supported by grants from the Deutsche Forschungsgemeinschaft, Vetenskaprådet, Åke Wiberg Foundation, and Loo&Hans Ostermans Foundation.
